# MAM-Mediated Mitochondrial Ca^2+^ Overload and Endoplasmic Reticulum Stress Aggravates Synaptic Plasticity Impairment in Diabetic Mice

**DOI:** 10.3390/brainsci15111157

**Published:** 2025-10-28

**Authors:** Jie Zhang, Jie Jiang, Haocong Li, Junliang Deng, Wei Dong, Huidan Deng

**Affiliations:** 1 College of Veterinary Medicine, Sichuan Agricultural University, Chengdu 611130, China; 15982356501@163.com (J.Z.); 17844611977@163.com (H.L.); dengjl213@126.com (J.D.); 2 Key Laboratory of Medical Electrophysiology, Ministry of Education & Medical Electrophysiological Key Laboratory of Sichuan Province, Institute of Cardiovascular Research, Southwest Medical University, Luzhou 646000, China; jiangjie2013@163.com; 3 Key Laboratory of Animal Diseases and Environmental Hazards of Sichuan Province, College of Veterinary Medicine, Sichuan Agricultural University, Chengdu 611130, China

**Keywords:** diabetes-associated cognitive dysfunction, mitochondria-associated endoplasmic reticulum membranes, endoplasmic reticulum stress, mitochondrial Ca^2+^ overload

## Abstract

**Background**: As a chronic threat to human and animal health, diabetes impairs cognition and synaptic plasticity through mechanisms that remain unresolved. This study aims to explore whether mitochondria-associated endoplasmic reticulum membrane (MAM)-mediated mitochondrial Ca^2+^ overload and endoplasmic reticulum stress plays an important role in high-glucose-induced synaptic plasticity damage in hippocampal neurons. **Methods and Results**: In diabetic mice, cognitive dysfunction was tightly linked to the synaptic plasticity impairment, manifesting as significant reductions in both mRNA and protein levels of PSD-95, GAP-43, and SYP. Concomitantly, aberrant increases in MAM number and structural alterations, along with pronounced up-regulation of Mfn2, were observed in hippocampal tissue from diabetic mice and cultured hippocampal neurons exposed to high glucose. High glucose also elevated MAM-located Ca^2+^ transporters (IP3R, GRP75, MCU, and VDAC1), provoking mitochondrial Ca^2+^ overload and activating ERS, particularly via the IRE1α pathway. Knockdown of Mfn2 ameliorated these high-glucose-induced MAM abnormalities, suppressed mitochondrial Ca^2+^ overload and ERS, and exerted a protective effect against high-glucose-induced synaptic plasticity damage. Application of the inhibitor MCU-i4 to block Ca^2+^ transport within MAM reduced high-glucose-induced mitochondrial Ca^2+^ overload, relieved ERS, and improved high-glucose-induced synaptic plasticity impairment. Application of the inhibitor 4μ8C to suppress the IRE1α pathway of ERS alleviated mitochondrial Ca^2+^ overload and improved high-glucose-induced synaptic plasticity impairment. **Conclusions**: High glucose elicits MAM dysregulation, which precipitates reciprocal mitochondrial Ca^2+^ overload and ER stress, jointly driving hippocampal synaptic plasticity impairment.

## 1. Introduction

Diabetes mellitus, one of the most severe metabolic disorders in humans globally [1], causes numerous complications such as retinopathy, renal failure, cardiovascular disease, and autonomic neuropathy [2]. Evidence indicates that diabetes also affects the central nervous system, particularly the hippocampus—a portion of the limbic system associated with cognitive activities—and leads to cognitive dysfunction in diabetic patients [3,4,5,6]. According to studies, diabetes increases the risk of cognitive dysfunction and dementia by 1.25 to 1.91 times [7], and approximately one out of every ten cases of dementia worldwide is associated with diabetes [8]. As of 2022, there were about 828 million adults with diabetes globally [9], and the majority of type 2 diabetes patients suffer from cognitive damage, with China accounting for 65%. As the world’s population ages, the prevalence of cognitive dysfunction caused by diabetes has increased significantly [10], posing a serious health risk and adding to the worldwide economic hardship.

Diabetic cognitive dysfunction is characterized by a deterioration in cognitive faculties such as learning, memory, and attention, primarily attributed to hippocampal neuronal damage [10]. Deficits in the structural integrity and functional efficacy of hippocampal synapses are a common feature and significant pathological manifestation of diabetic cognitive dysfunction, characterized by alterations in the morphological indices of hippocampal synaptic plasticity [11,12,13,14] and adverse impacts on synaptic transmission [15,16,17,18]. Researchers have utilized fluoxetine [19] and artemisinin [20] to mitigate cognitive dysfunction in AD and T2DM mice by reducing Aβ1-40 and Aβ1-42 levels in the hippocampus and activating the hippocampal PI3K/Akt pathway, respectively, thereby enhancing hippocampal neuronal synaptic plasticity. However, the precise mechanisms underlying diabetic cognitive dysfunction remain elusive.

Mitochondria-associated endoplasmic reticulum membranes (MAMs), representing areas of intimate contact between the mitochondria and the endoplasmic reticulum (ER), are extensively involved in a variety of physiological processes and play a crucial role in the maintenance of cellular homeostasis [21,22,23]. Mfn2, which localizes to both the ER and mitochondria, has been identified as a connector within the MAM structure [24]. Preliminary studies have indicated that Mfn2 depletion results in a reduction in MAMs [24], while its overexpression leads to an increase [25]. Meanwhile, mitochondria and ER are highly enriched in Ca^2+^-transport proteins, which made MAMs act as critical Ca^2+^ homeostatic regulators. Ca^2+^ is first discharged from the ER through inositol-1,4,5-trisphosphate receptors (IP3Rs) or ryanodine receptors (RyRs) [26]. This efflux is funneled to mitochondria via a macromolecular complex in which IP3R/RyR is tethered to the outer membrane voltage-dependent anion channel (VDAC1) by glucose-regulated protein 75 (GRP75) [27]. Ca^2+^ entry into the matrix is subsequently gated by the mitochondrial Ca^2+^ uniporter (MCU) complex [21]. Thus, the IP3R-GRP75-VDAC1-MCU and RyR-VDAC1-MCU assemblies constitute the core Ca^2+^-translocating machinery within MAM that synchronize ER Ca^2+^ release with mitochondrial Ca^2+^ uptake.

A growing body of evidence links disturbed Ca^2+^ homeostasis to diabetes-associated cognitive dysfunction. Singhal K and Sandhir R [28] reported elevated Cav1.2 mRNA and protein levels in the brains of diabetic mice, leading to exaggerated synaptic Ca^2+^ influx; the L-type Ca^2+^ channel blocker nimodipine normalized Cav1.2 expression, restored Ca^2+^-dependent synaptic plasticity, and reversed cognitive deficits. Similarly, high glucose up-regulates STIM1, promoting apoptosis-driven neuronal injury and cognitive dysfunction in diabetic rats. Attenuating Ca^2+^ influx markedly mitigates this damage [29]. The scientific community has recently shifted its focus toward elucidating the mechanisms of MAM-mediated Ca^2^dyshomeostasis. Hutchins et al. [30] demonstrated that disrupting MAM-dependent ER-to-mitochondria Ca^2+^ transfer compromises synaptic plasticity and slows axonal growth. Under hypoxia or ERS, MAM Ca^2+^ transfer is derailed, triggering mitochondrial Ca^2+^ overload, cytochrome C release, and apoptotic neuronal loss that exacerbates functional deficits [31]. Chang et al. [32] further showed that in neuroblastoma cells, high-glucose-induced injury is accompanied by aberrant MAM formation; SIRT3 curtails these ectopic contacts, mitigates mitochondrial Ca^2+^ overload, and protects neurons. However, whether MAM-mediated Ca^2+^ dyshomeostasis contributes to high-glucose-induced hippocampal synaptic plasticity deficits remains undefined.

Ca^2+^ dyshomeostasis also perturbs ER function, provoking endoplasmic reticulum stress (ERS) and activating the unfolded protein response (UPR) [33], which involves the PERK, IRE1α, and ATF6 pathways [34,35]. Treatment with SERCA inhibitors such as thapsigargin (TG) elicits ER Ca^2+^ leak, depletes luminal Ca^2+^ stores, and thereby evokes ER dysfunction and activates the UPR [36]. Conversely, the VMP1 protein binds and positively regulates SERCA [37]; VMP1 loss selectively activates PERK while suppressing IRE1α and ATF6 signaling [38]. Many ER-located chaperones including calnexin [39], calreticulin [40], and Grp94 [41], contain low-affinity Ca^2+^-binding sites and is Ca^2+^-sensitive. Meanwhile, ERS is a well-established driver of synaptic pathology. It promotes Aβ oligomer formation and tau hyperphosphorylation [42,43], α-synuclein aggregation [44], and impaired autophagic clearance of mutant huntingtin [45], culminating in synaptic loss, plasticity failure, and neuronal death that underlie cognitive decline. He et al. [46] showed that zonisamide elevates Hrd1, suppresses ERS, reduces tau Ser396/404 hyperphosphorylation and cortical Aβ deposition, and rescues synaptic function in diabetic mice. Taken together, these findings raise the possibility that high-glucose-induced Ca^2+^ dyshomeostasis activates ERS, which in turn amplifies neuronal and synaptic injury. Yet it remains unknown how MAM-mediated Ca^2+^ overload synergizes with ERS to trigger hippocampal synaptic plasticity deficits under high-glucose conditions.

In summary, cognitive dysfunction is a prominent diabetic complication in which synaptic plasticity deficits are critical. Although researchers have confirmed that MAM alterations under high glucose are linked to this damage, it remains unclear whether MAM-mediated Ca^2+^ imbalance drives the injury and how ER stress is involved. To address these questions, we established a mouse model of diabetes-associated cognitive impairment and a complementary in vitro model of high-glucose-induced hippocampal synaptic plasticity impairment, and found that under high-glucose conditions, Mfn2-regulated MAM architectural and numerical abnormalities provoke hippocampal plasticity impairment. Interference with MCU and IRE1α further demonstrated that mitochondrial Ca^2+^ overload and ERS synergistically exacerbate synaptic damage during this process. This research lays a theoretical foundation for the identification of therapeutic targets for cognitive dysfunction in diabetes.

## 2. Materials and Methods

### 2.1. Animals and Drug Administration

Sixty SPF-grade KM male mice (eight-week-old) were purchased from Sichuan Dashuo Biological Co., Ltd. (Chengdu, China). The mice were housed under standard laboratory conditions with an artificial 12 h light/12 h dark cycle and were fed adaptively for one week before the experiments commenced.

Then, the mice were randomly divided into two groups and housed with three or two mice per cage. To establish a type 2 diabetes mellitus (T2DM) mouse model, thirty mice were fed a high-sugar, high-fat diet for six weeks to induce insulin resistance. Subsequently, the mice were administered streptozotocin (STZ, 30 mg/kg/day, Sigma-Aldrich (St. Louis, MO, USA, S0130), dissolved in citrate buffer (0.1 M, pH = 4.5), via intraperitoneal injection for five consecutive days [47]. Control group mice were fed a regular diet and received an equivalent volume of sodium citrate buffer. Two weeks post-injection, blood glucose levels were measured from the tail tip. Mice were considered diabetic when fasting blood glucose reached ≥11.1 mmol/L. Six weeks after the final STZ injection, they were subjected to behavioral tests and molecular analyses. Before tissue collection, animals were anesthetized with 2–3% isoflurane in oxygen and euthanized by cervical dislocation.

Each mouse was utilized in the current experiment. This experimental protocol obtained approval from the Animal Care and Use Committee of Sichuan Agricultural University (Chengdu, China Approval No. 20230677).

### 2.2. Behavioral Tests

The Morris water maze (MWM) test is used to assess the cognitive abilities of animals.

Over the first four days, mice are subjected to daily trials to identify a submerged platform that acts as an escape route. On the fifth day, we remove the platform and measure how much time the mouse spends in the region where the platform used to be, how far it swims, and how many times it crosses the spot where the platform was, in order to assess spatial learning and memory function.

### 2.3. Golgi Staining and Spine Analysis

The brain tissue of the mice was fixed and hardened with potassium dichromate, followed by silver plating using silver nitrate solution. Section, rinse, dehydrate, and clarify the tissue before mounting for microscopic observation (3DHISTECH, Pannoramic MIDI, Budapest, Hungary). For each brain sample, five sections were imaged to ensure representative data. ImageJ Win64 was used to automatically quantify dendritic spine density by setting fluorescence intensity thresholds and counting spines along defined dendritic segments, and to analyze dendritic spine distribution and density to assess neuronal connectivity and synaptic activity. The protocol followed the methods of Zhu Song et al. [48].

### 2.4. Nissl Staining

The brain tissue was first fixed in a 10% formalin solution for 24 h, followed by rinsing in distilled water, and then stained with cresyl violet. After staining, the sections were dehydrated through a graded series of ethanol, cleared in xylene, and cover slipped with a resinous mounting medium. For each brain sample, five sections were imaged to ensure representative data. The sections were dried and examined under an upright optical microscope (NIKONECLIPSEE100, Nikon Corporation, Tokyo, Japan). To quantify the staining, Nissl-stained neurons were manually counted in three 200 × 200 µm fields per region. The average density (cells/mm^2^) was calculated for every mouse. Values were then normalized to the mean density of the CK (set to 100%) and expressed as % of CK (mean ± SD).

### 2.5. Quantitative Real-Time PCR (qRT-PCR)

RNA extraction kit (Accurate Biology, Changsha, China, AG21017) was chosen to extract total RNA according to the manufacturer’s protocol. cDNA synthesis was made with RNA in combination with the reverse transcription kit (Accurate Biology, China AG11707) in accordance with the corresponding specifications. The gene data came from the National Center for Biotechnology Information (NCBI), and Sangon Biotech (Shanghai, China) took charge of primer design and synthesis. Table 1 shows the primer sequences.

mRNA expression was assessed in a 20 µL volume using SYBRPRIME qPCR kit (Accurate Biology, China AG11701) according to the corresponding specifications. Cycling conditions were as follows: 95 °C for 30 s (one cycle), followed by 40 cycles of 95 °C for 5 s and 60 °C for 30 s. Subsequently, melt curves were investigated to identify PCR specificity. Additionally, the results of qRT-PCR were analyzed using the 2^−ΔΔCT^ method [49].

### 2.6. Cell Culture and Treatments

In this experiment, primary extracted and stably cultured rat hippocampal neuron cells were used, cultured in DMEM medium (Boster, Wuhan, China, PYG0073) containing 10% fetal bovine serum and placed in a constant temperature incubator at 37 °C with 5% carbon dioxide, with regular medium changes and passaging.

To explore the effects of a high-glucose environment on the structure of MAM and synaptic plasticity in hippocampal neurons, we set up four experimental groups and treated them with different concentrations of glucose (0, 25, 50, and 100 mM/L) for 48 h to collect cell samples.

To investigate the impact of Mfn2-related high glucose on the structure of MAM and synaptic plasticity in hippocampal neurons, we employed siRNA technology to knockdown the expression of the Mfn2 gene in cells. Si-Mfn2 and RNAfit (Hanheng Biotechnology Co., Ltd., Shanghai, China, HB-RF-1000) were used in combination to treat cells for 60 h. We set up a blank control group, a negative control group, a high-glucose group (100 mM/L, 48 h), a Si-Mfn2 group, and a co-treatment group of high glucose and Si-Mfn2 group.

We employed 5 μM MCU-i4 (MCE, Shanghai, China, HY-138620) to suppress MCU protein expression and 5 μM 4μ8C (Selleck, Shanghai, China, S7272) to inhibit IRE1α phosphorylation. First, the concentration range of each inhibitor was preliminarily defined by CCK-8 assays for cell viability. Cells were then seeded in plates; after attachment, the indicated concentrations of MCU-i4 or 4μ8C were added. Following 48 h of treatment, cells were harvested for protein extraction to assess inhibitor efficacy and to perform downstream analyses.

### 2.7. Transmission Electron Microscopy (TEM)

Cell and tissue samples were fixed with 2.5% glutaraldehyde at 4 °C overnight and then post-fixed with 1% osmium tetroxide for 2 h. After rinsing with PBS buffer, the samples were dehydrated using an ethanol gradient, infiltrated with 100% propylene oxide, and embedded in epoxy resin for 24 h. Ultrathin sections (50 nm) were prepared and mounted on copper grids. For each sample, eight ultrathin sections were imaged to ensure comprehensive data collection. Finally, the structures and quantities of MAM and synapses were observed using a transmission electron microscope (JEOL, Tokyo, Japan, JEM-2100F). The number and size of MAM and synapses were manually quantified by measuring the area and counting the structures in defined fields of view using ImageJ software.

### 2.8. Western Blot

Extract hippocampal neuron cells and tissue proteins according to the Protein Extraction Kit (Beyotime, Shanghai, China, R0018M). After lysing and centrifuging the samples, collect the supernatant for total protein and quantify the protein using the BCA Assay Kit (Beyotime, China P0011). Subsequently, 15 μg of protein from each sample was loaded and separated by 12% SDS-PAGE, followed by transfer to a PVDF membrane. Afterwards, 5% non-fat milk dissolved in PBST was employed for membrane blocking, followed by incubation by using primary antibodies (overnight at 4 °C) and HRP-labeled secondary antibodies (1 h at room temperature) successively. The ECL kit (Oriscience, Shanghai, China PD203) was used in visualization. The protein bands were visualized with Touch Imager System (E-BOLT, Shenzhen, China). Finally, significant differences in protein expression were analyzed with ImageJ software. The RRIDs of the antibodies used in Western blotting are shown in Table 2.

### 2.9. Fluorescent Colocalization Analysis

Cells were grown on coverslips. Following the designated treatments, cells were rinsed three times with serum-free DMEM. Mito-Tracker Red CMXRos (Beyotime, China C1035) and ER-Tracker Green (Beyotime, China C1042) were applied to stain the cells for 30 min each, highlighting the mitochondria and ER. For mitochondrial Ca^2+^ imaging, we chose Rhod-2/AM (Yeasen, Shanghai, China, HB230423) and Mito-Tracker Green (Beyotime, China C2005) to stain the cells for 15–60 min each. For MCU–p-IRE1α colocalization, primary antibodies against MCU (HUABIO, Wuhan, China, HA723565) and p-IRE1α (HUABIO, China HA721980) were incubated for 20–40 min each. The cells were then mounted using a DAPI-containing mounting medium (Beyotime, China P0131).

For each experimental condition, 10 fields of view were imaged to ensure representative data. The colocalization of mitochondria and ER was examined under a fluorescence microscope (Zeiss Axiovert 5 Digital, Carl Zeiss AG, Jena, Germany). The degree of colocalization was assessed by evaluating the association between ER and mitochondrial fluorescence signals.

### 2.10. Flow Cytometry

Collect the cell suspension, wash once with PBS, and save the pellet. Dilute Rhod-2/AM (Yeasen, China HB230423) 1:200 in PBS to prepare a 5 μM working solution. Incubate the cells in this solution at 37 °C for 40 min, inverting every 3–5 min to ensure complete probe loading. After incubation, centrifuge and discard the supernatant. Wash the cells three times with PBS, resuspend, and analyze immediately by flow cytometry. Data were processed using CytExpert 2.0 software.

### 2.11. Statistical Analysis

The results were illustrated as mean ± SD. Normality and variance homogeneity were assessed using Shapiro–Wilk test and Levene’s test, respectively. Comparisons between the control and experimental groups were analyzed by one-way ANOVA followed by Dunnett’s post hoc test. *p* < 0.05 suggested that the value was statistically significant.

## 3. Results

### 3.1. Hyperglycemia-Induced Neuronal and Synaptic Damage Contribute to Cognitive Dysfunction in Diabetic Mice and Hippocampal Neurons

**Figure 1:**
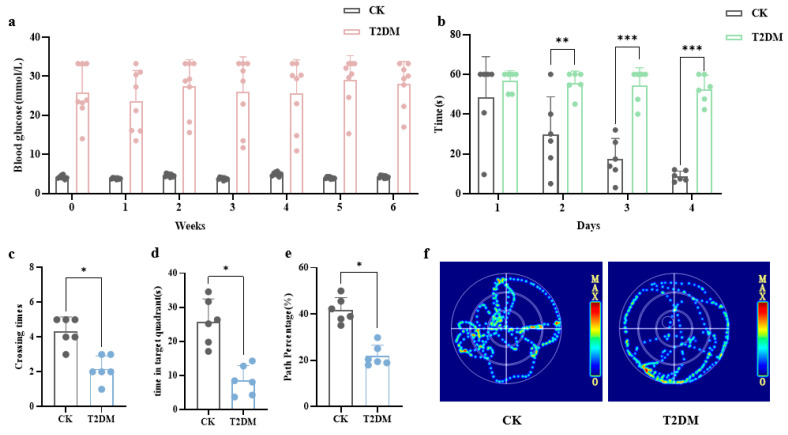
Cognitive dysfunction in diabetic mice. (**a**) Fasting blood glucose levels of mice. (**b**) Escape latency in the Morris water maze test. (**c**) Number of platform crossings in the spatial probe test. (**d**) Time spent in the target quadrant during the spatial probe test. (**e**) Percentage of distance traveled in the target quadrant during the spatial probe test. (**f**) Representative swimming paths during the test phase. Data are presented as mean ± standard deviation. * *p* < 0.05, ** *p* < 0.01, *** *p* < 0.001, compared with the control group.

To verify the effectiveness of the diabetes model, we conducted blood glucose tests. The results showed that all mice given STZ along with a high-sugar, high-fat diet had fasting blood sugar levels over 11.1 mmol/L (Figure 1a), making them ready for the next round of experiments.

To investigate the cognitive functions of diabetic mice, we conducted the MWM test, the results of which are shown in Figure 1. Starting from the second day of the experiment, the time taken by the T2DM group to find the platform (escape latency) was significantly longer than that of the control group, and a highly significant difference was observed between the two groups on the third and fourth days (*p* < 0.01, *p* < 0.001) (b). In the 60 s spatial exploration test, the T2DM group had fewer crossings of the platform (c), less time spent in target quadrant (d), and a lower percentage of total distance traveled in target quadrant (e) compared to the control group, with significant differences (*p* < 0.05). These findings indicate cognitive dysfunction in diabetic mice.

**Figure 2:**
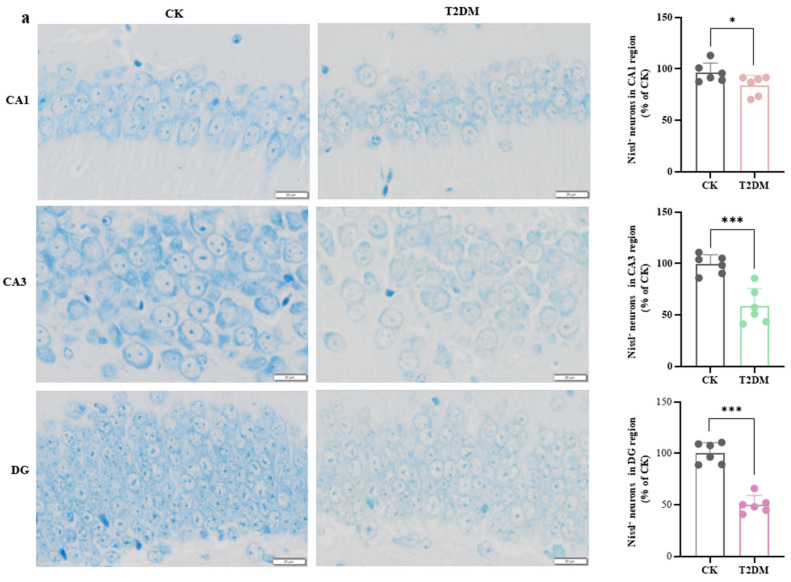

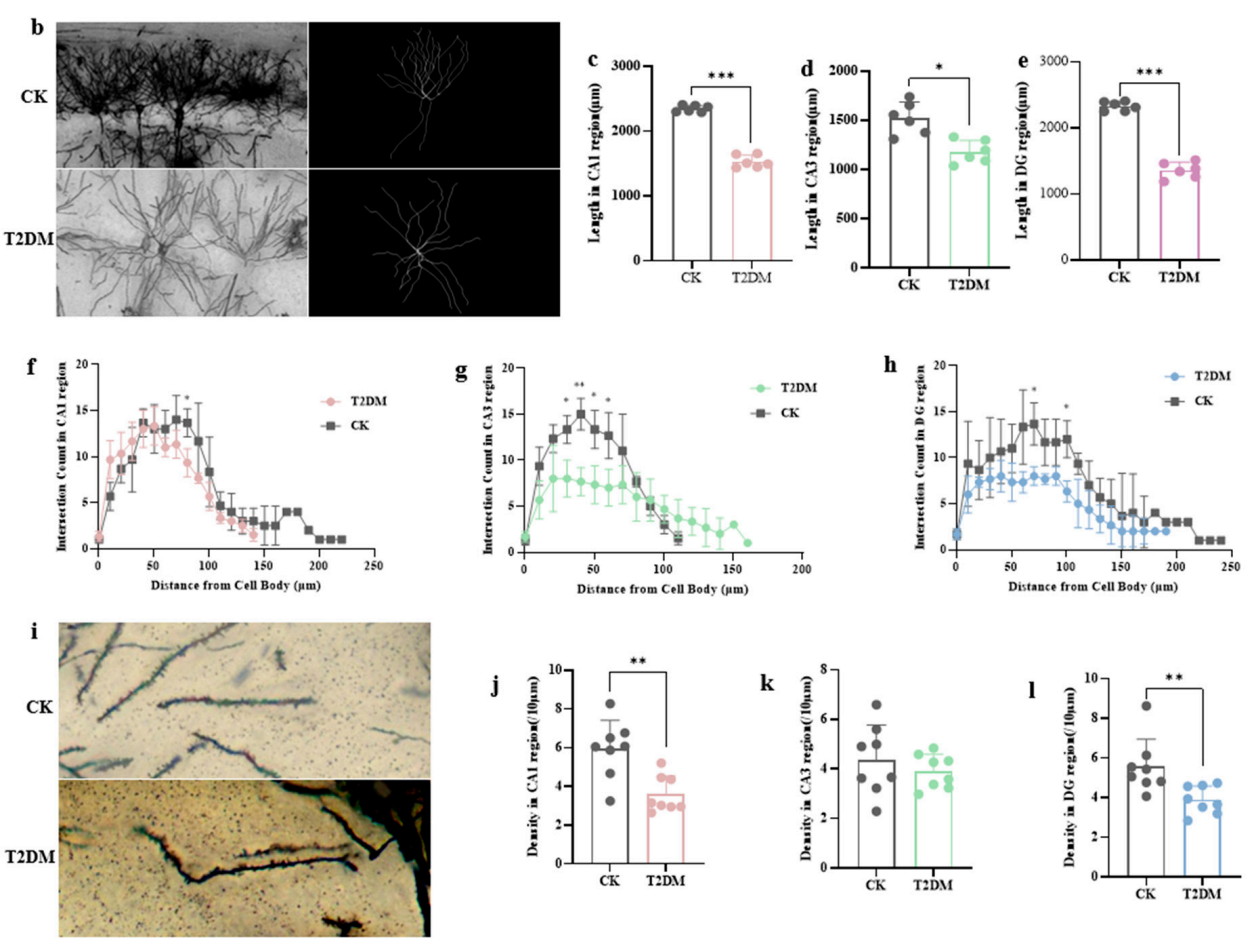
Neuronal and synaptic damage in the hippocampus of diabetic mice. (**a**) Nissl staining analysis of hippocampal neuronal loss in diabetic mice. (**b**) Neuronal tracing images of the T2DM group and CK. (**c**–**e**) Total dendritic length in the CA1, CA3, and DG region. (**f**–**h**) Dendritic complexity in the CA1, CA3, and DG region. (**i**) Dendritic spines captured under high magnification. (**j**–**l**) Dendritic spine density in the CA1, CA3, and DG region. Data are presented as mean ± standard deviation. * *p* < 0.05, ** *p* < 0.01, *** *p* < 0.001, compared with the control group.

To assess hippocampal neuronal damage in diabetic mice, we employed Nissl staining and Golgi staining techniques. Figure 2a showed a reduction in Nissl bodies in the hippocampal tissue of diabetic mice through Nissl staining, with the most pronounced neuronal loss occurring in the CA3 and DG regions.

Following Golgi staining, neuronal images were processed in ImageJ and then traced and analyzed using Neuron J to obtain dendritic lengths, as depicted in Figure 2b. The total dendritic length in the CA1 region (c), CA3 region (d), and DG region (e) of T2DM mice was significantly shorter than that of the control group, with varying degrees of difference in each area (*p* < 0.05, *p* < 0.001).

Sholl’s concentric circle analysis was performed on the traced neuronal images to determine the complexity of dendritic trees at different distances from the cell body. In the CA1 region (f), the number of intersections of dendrites with concentric circles was lower in the T2DM group compared to the control group (CK), with significant differences at 60 µm and beyond (*p* < 0.05). In the CA3 region (g), the number of intersections was lower in the T2DM group within 0–80 µm from the cell body, with significant differences at 30 µm, 40 µm, 50 µm, and 60 µm, and the maximum dendritic length in the T2DM group was shorter than in the CK (*p* < 0.05, *p* < 0.01). In the DG region (h), the number of intersections was lower in the T2DM group, with significant differences at 80 µm and 100 µm, and the maximum dendritic length in the T2DM group was shorter than in the CK (*p* < 0.05).

Figure 2i shows dendritic spines captured at high magnification, and the density of dendritic spines was analyzed after processing the captured spines with ImageJ. The density of dendritic spines in the CA1 region (j), CA3 region (k), and DG region (l) of the T2DM group was lower than that of the CK, with varying degrees of difference in each area.

**Figure 3:**
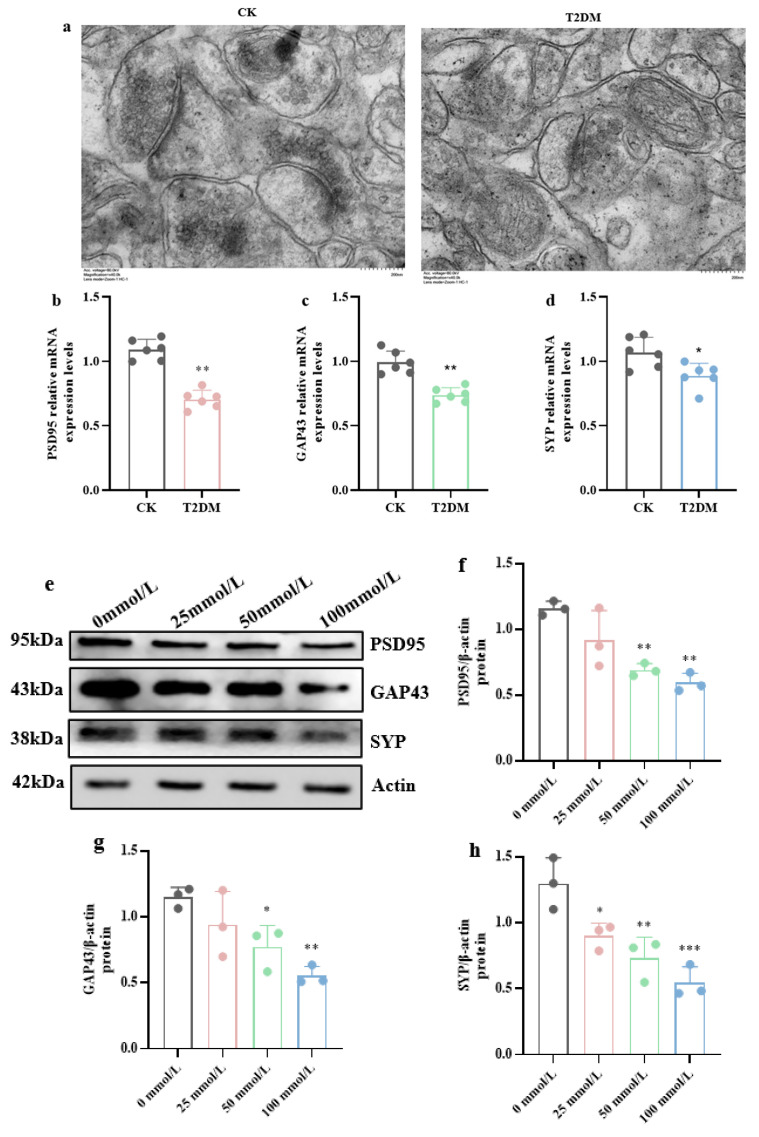
Synaptic damage in the hippocampus of diabetic mice and hippocampal neurons exposed to high glucose. (**a**) Synaptic structure in the hippocampus of mice detected by electron microscopy. (Scale bar = 200 nm) (**b**–**d**) Relative mRNA expression levels of PSD95, GAP43, and SYP in the hippocampal tissue of mice. (**e**) Western blot analysis of synaptic protein expression in hippocampal neurons exposed to varying high glucose. (**f**–**h**) Expression levels of PSD95, GAP43, and SYP protein in hippocampal neuronal cells. Data are presented as mean ± standard deviation. * *p* < 0.05, ** *p* < 0.01, *** *p* < 0.001, compared with the control group.

Then, TEM and qRT-PCR were employed to investigate synaptic damage in the hippocampus. Observation of hippocampal tissue sections under electron microscopy revealed that the synaptic structure in the CK was normal and clear, while in the T2DM group, the synaptic structure was less distinct, with widened synaptic clefts, a reduced number of synaptic vesicles, sparse distribution, and a blurred structure (Figure 3a).

Fluorescent quantitative PCR results on hippocampal tissue (Figure 3b–d) showed that the mRNA expression levels of postsynaptic density protein 95 (PSD-95), growth-associated protein 43 (GAP-43), and synaptophysin (SYP) were significantly decreased in the T2DM group compared to the CK (*p* < 0.05, *p* < 0.01), indicating that diabetes causes synaptic plasticity damage in the hippocampus of mice.

Similarly, in hippocampal neuronal cells cultured in vitro, high-glucose treatment led to a decrease in the expression of PSD-95, GAP-43, and SYP in the total protein of hippocampal neuronal cells, indicating that high glucose causes synaptic plasticity damage.

### 3.2. High-Glucose-Induced MAM Dysfunction, Mitochondrial Ca^2+^ Overload, and ER Stress in Hippocampal Cells

**Figure 4:**
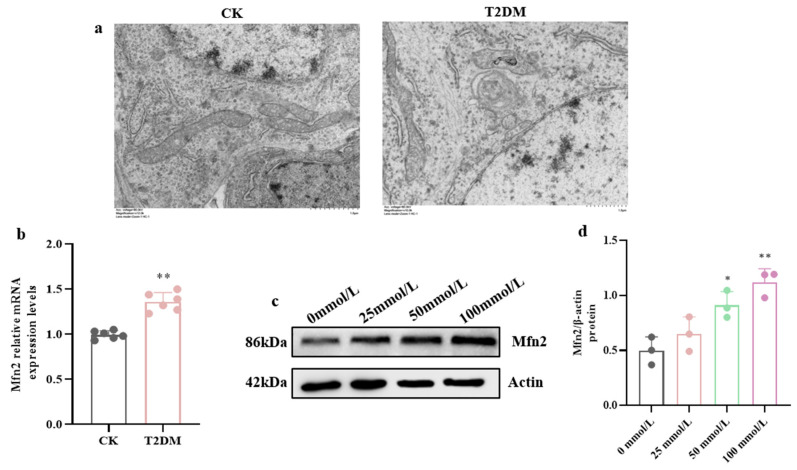
High-glucose-induced MAM structural and Mfn2 expression abnormalities. (**a**) Electron microscopic observation of MAM structure in mouse hippocampal cells. (Scale bar = 1.0 μm) (**b**) Relative mRNA expression levels of Mfn2 in the hippocampus of diabetic mice. (**c**,**d**) Protein expression levels of Mfn2 in hippocampal neuronal cells. Data are presented as mean ± standard deviation. * *p* < 0.05, ** *p* < 0.01, compared with the control group.

We observed the MAM structure in mouse hippocampal cells under electron microscopy (Figure 4a) and found that in the CK, the mitochondrial and ER structures were intact and clear. In contrast, in the T2DM group, the mitochondrial cristae were blurred, the matrix was lost, the ER was swollen, and the contact area between mitochondria and ER increased, with a higher number of MAM and longer functional regions.

Interestingly, according to the results of qRT-PCR and WB, we found that Mfn2 expression was significantly up-regulated in the hippocampal tissues of diabetic mice (Figure 4b) and in hippocampal neurons exposed to high glucose (Figure 4c,d).

**Figure 5:**
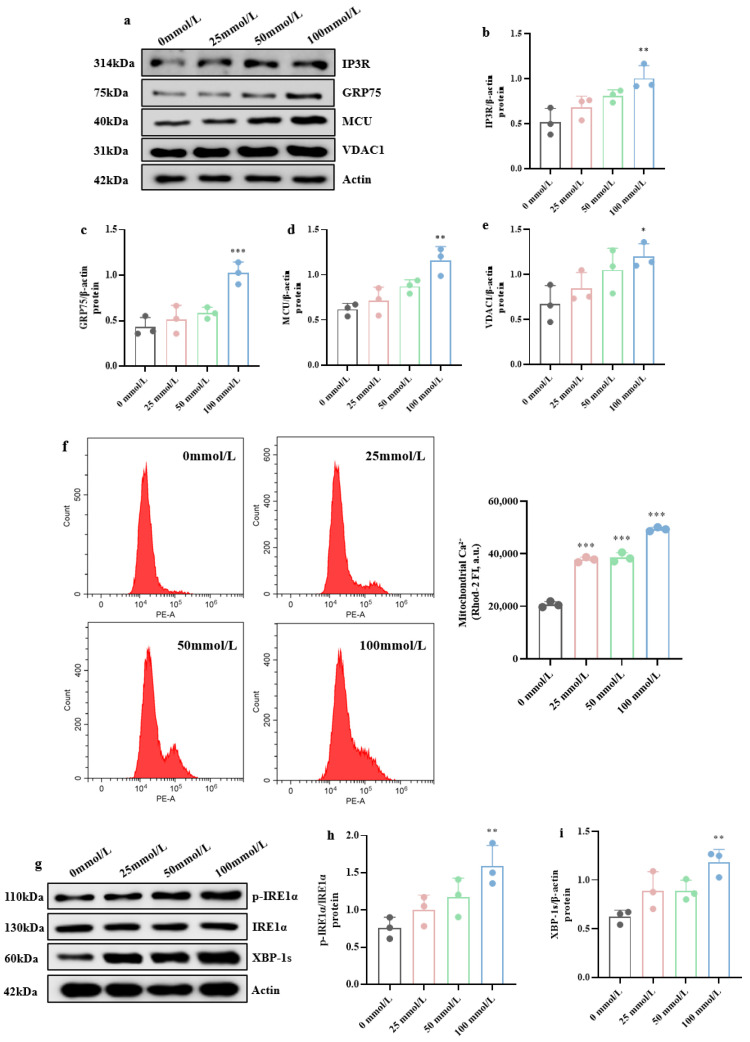
Mitochondrial Ca^2+^ overload and ERS induced by high glucose. (**a**) Western blot results of Ca^2+^-transport protein expression. (**b**–**e**) Expression levels of IP3R, GRP75, MCU, and VDAC1 protein in hippocampal neuronal cells. (**f**) The relative mitochondrial Ca^2+^ amounts determined by flow cytometry. (**g**) Western blot results of ERS-related protein expression. (**h**,**i**) Quantification of p-IRE1α/IRE1α and XBP-1s protein expression. Data are presented as mean ± standard deviation. * *p* < 0.05, ** *p* < 0.01, *** *p* < 0.001, compared with the control group.

Simultaneously, Western blotting (Figure 5a–e) revealed elevated expression of MAM-localized Ca^2+^-transport proteins under high glucose: GRP75 was markedly increased (*p* < 0.001), IP3R and MCU were significantly up-regulated (*p* < 0.01), and VDAC1 was elevated (*p* < 0.05), indicating disrupted Ca^2+^ homeostasis at MAM. Flow cytometry (Figure 5f) further showed that mitochondrial Ca^2+^ levels were profoundly higher in the high-glucose group than in control group (*p* < 0.001), confirming high-glucose-induced mitochondrial Ca^2+^ overload.

We next examined ER stress by Western blot. Treatment with 100 mmol/L glucose markedly increased IRE1α phosphorylation (*p* < 0.01) and the relative abundance of the active spliced form of XBP-1 (XBP-1s, *p* < 0.01) (Figure 5g–i), demonstrating that high glucose evokes ER stress in hippocampal neurons.

### 3.3. MAM-Mediated Mitochondrial Ca^2+^ Overload and ERS Drive High-Glucose-Induced Synaptic Plasticity Injury in Hippocampal Cells

**Figure 6:**
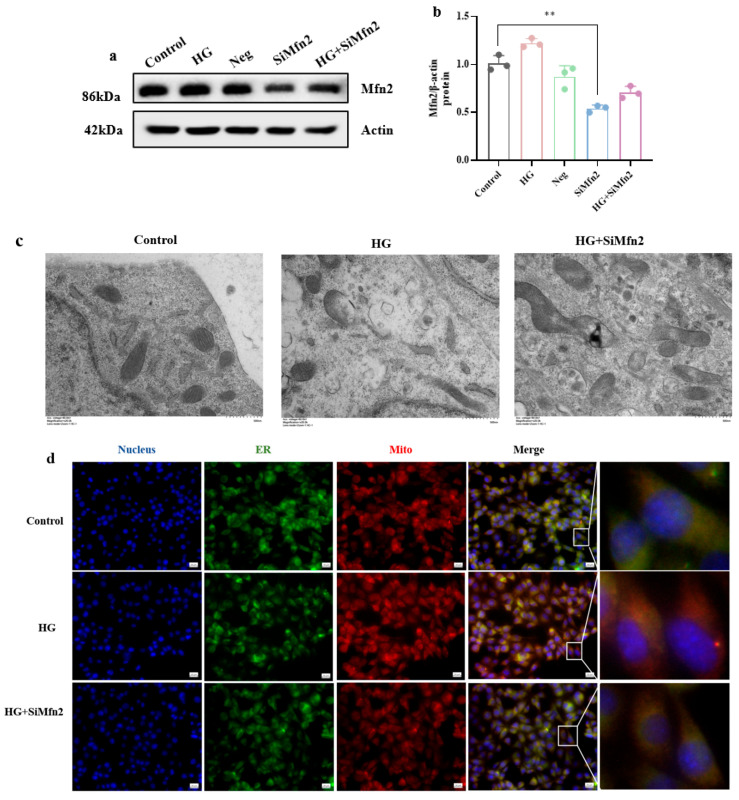
Mfn2 knockdown rectifies MAM dysfunction induced by high glucose. (**a**,**b**) Validation of Mfn2 knockdown efficiency. (**c**) Improvement in mitochondrial and ER damage and MAM abnormalities after Mfn2 knockdown under high-glucose conditions. (Scale bar = 1.0 μm) (**d**) Fluorescence colocalization showing increased MAM in hippocampal neuronal cells exposed to high glucose and improvement after Mfn2 knockdown. (Scale bar = 20 μm) (*n* = 3). Data are presented as mean ± standard deviation. ** *p* < 0.01.

To explore the role of Mfn2-mediated MAM in synaptic damage in hippocampal neuronal cells exposed to high glucose, we knocked down the expression of Mfn2 in hippocampal neuronal cells. Figure 6a,b show that Mfn2 expression was greatly reduced after gene knockdown, as confirmed by Western blotting (*p* < 0.01).

Consistent with in vivo TEM findings in Figure 4a, MAM structural and functional abnormalities were also observed in hippocampal neuronal cells cultured in vitro. Knocking down Mfn2 improved the mitochondrial and ER damage and the abnormal increase in MAM caused by high glucose (Figure 6c). We also used fluorescence colocalization to observe that in hippocampal neuronal cells exposed to high glucose, the number of colocalization points between mitochondria and ER increased, indicating an abnormal increase in MAM, which was improved by Mfn2 knockdown (Figure 6d).

**Figure 7:**
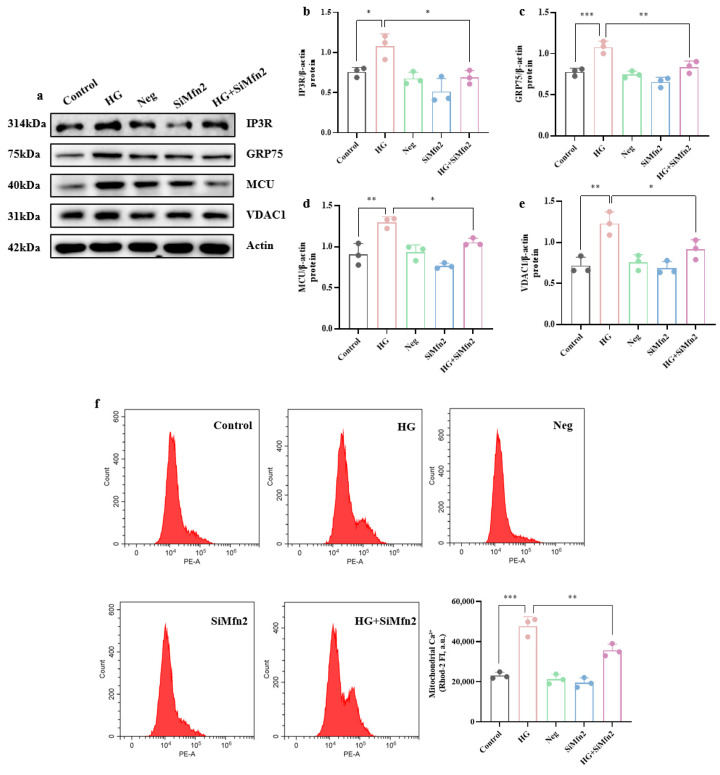

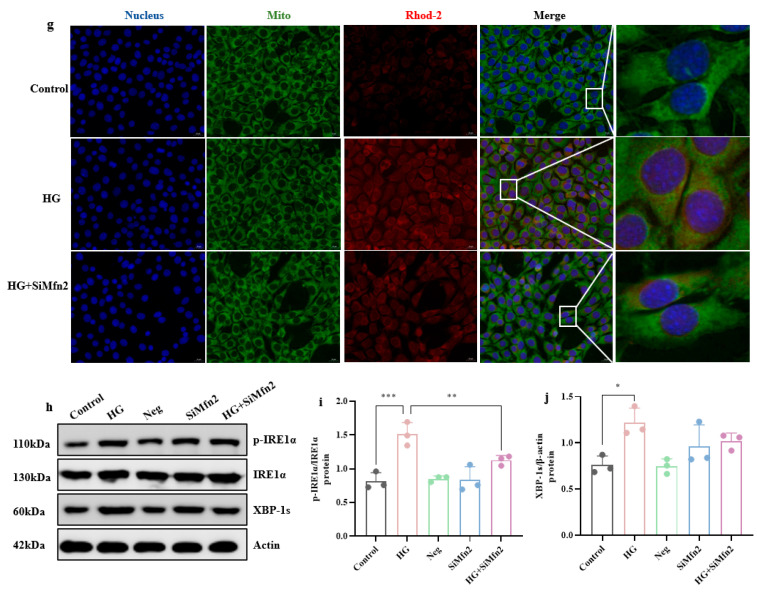
Mfn2-mediated MAM alleviates high-glucose-induced Ca^2+^ dyshomeostasis and ERS. (**a**) Western blot results of Ca^2+^-transport protein expression. (**b**–**e**) Expression levels of IP3R, GRP75, MCU, and VDAC1 protein in hippocampal neuronal cells. (**f**) Relative mitochondrial Ca^2+^ amounts determined by flow cytometry. (**g**) Fluorescence showing increased mitochondrial Ca^2+^ amounts in hippocampal neuronal cells exposed to high glucose and improvement after Mfn2 knockdown. (Scale bar = 20 μm) (*n* = 3). (**h**) Western blot results of ERS-related protein expression. (**i**,**j**) Quantification of p-IRE1α/IRE1α and XBP-1s protein expression. Data are presented as mean ± standard deviation. * *p* < 0.05, ** *p* < 0.01, *** *p* < 0.001.

Western blotting also showed that Mfn2 knockdown attenuated the up-regulation of MAM-localized Ca^2+^-transport proteins induced by high glucose (Figure 7a–e): GRP75 was markedly restored (*p* < 0.01), and IP3R, MCU, and VDAC1 were significantly normalized (*p* < 0.05). Using Rhod-2 AM, we confirmed by flow cytometry (Figure 7f) and immunofluorescence (Figure 7g) that Mfn2 knockdown alleviated high-glucose-induced mitochondrial Ca^2+^ overload.

Figure 7d–f also show that Mfn2 knockdown markedly attenuated high-glucose-induced IRE1α phosphorylation (*p* < 0.01), thereby alleviating endoplasmic reticulum stress in hippocampal neurons.

**Figure 8:**
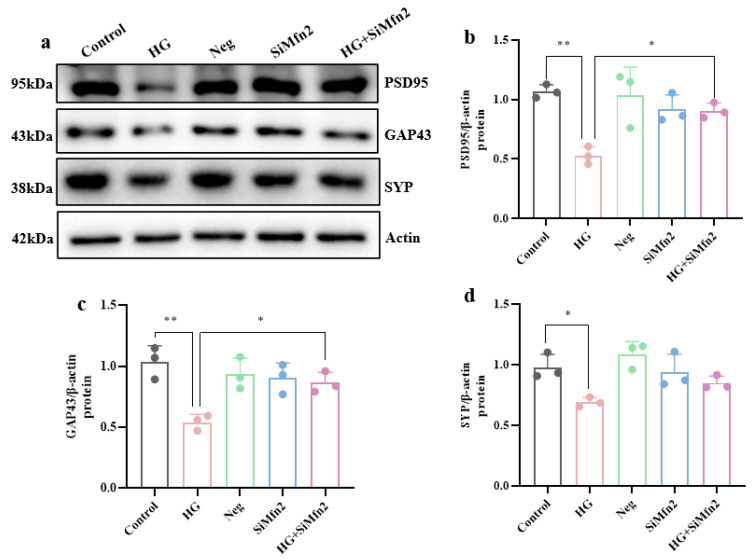
Mfn2 knockdown alleviates high-glucose-induced hippocampal synaptic plasticity damage. (**a**) Expression levels of synaptic-related proteins. (**b**–**d**) Expression levels of PSD95, GAP43, and SYP protein in hippocampal neuronal cells. Data are presented as mean ± standard deviation. * *p* < 0.05, ** *p* < 0.01.

Then, to investigate whether Mfn2 mediates the abnormal increase in MAM to promote high-glucose-induced synaptic plasticity damage, we used Western blotting to detect the expression of synaptic plasticity-related proteins in hippocampal neuronal cells after high-glucose treatment combined with Mfn2 knockdown. The results showed (Figure 8a–d) that Mfn2 knockdown could improve high-glucose-induced synaptic plasticity damage, shown as the increased protein expression of PSD95 and GAP43 compared to the HG group (*p* < 0.05).

### 3.4. High-Glucose-Induced ERS and Mitochondrial Ca^2+^ Overload Synergistically Impair Hippocampal Synaptic Plasticity

**Figure 9:**
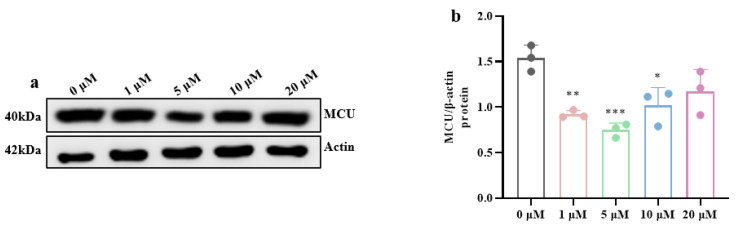

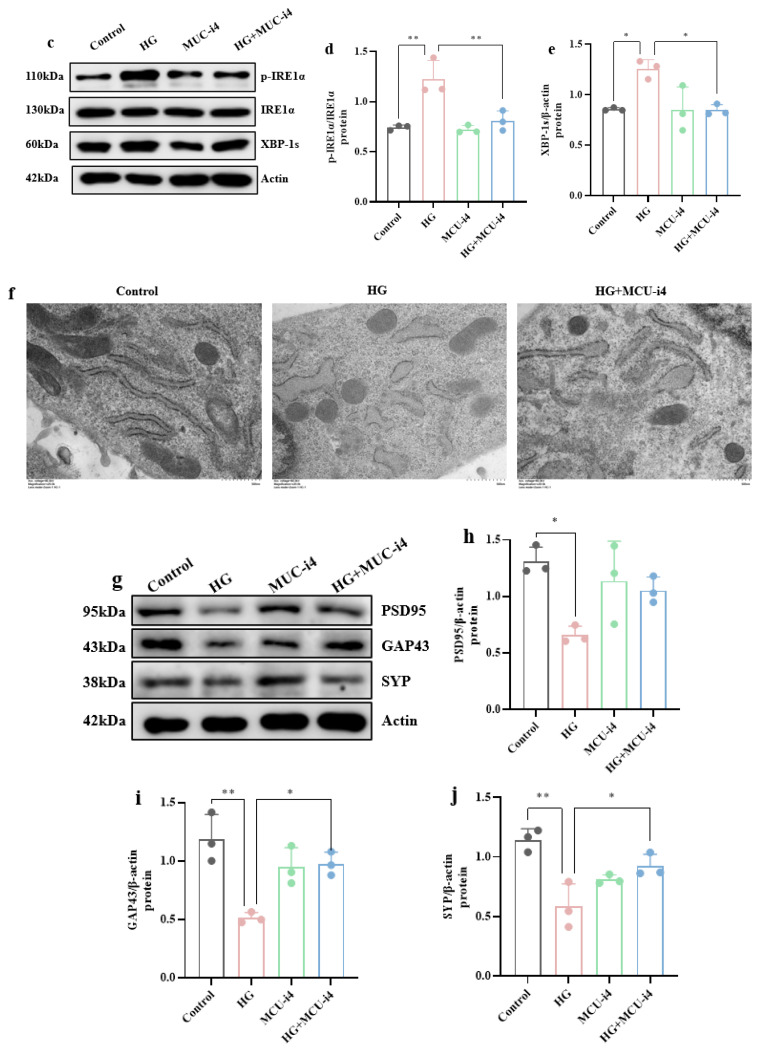
MCU inhibition attenuates high-glucose-induced ERS. (**a**) Western blot results of MCU protein expression. (**b**) Quantification of MCU protein expression. (**c**) Western blot results of ERS-related protein expression. (**d**,**e**) Quantification of p-IRE1α/IRE1α and XBP-1s protein expression. (**f**) Improvement in ERS after suppressing the expression of MCU protein in high-glucose conditions. (Scale bar = 1.0 μm) (**g**) Expression levels of synaptic-related proteins. (**h**–**j**) Expression levels of PSD95, GAP43, and SYP protein in hippocampal neuronal cells. Data are presented as mean ± standard deviation. * *p* < 0.05, ** *p* < 0.01, *** *p* < 0.001.

To examine whether high-glucose-induced ERS is mechanistically linked to mitochondrial Ca^2+^ overload, we selectively attenuated Ca^2+^ flux at MAM by using the MCU blocker MCU-i4. Western blotting (Figure 9a,b) demonstrated that 5 μM MCU-i4 potently suppressed MCU protein expression (*p* < 0.001). Then, Western blotting (Figure 9c–e) revealed that suppressing the expression of MCU protein markedly alleviated high-glucose-triggered ERS (*p* < 0.01, *p* < 0.05). Ultrastructural analysis of transmission electron microscopy (Figure 9f) further showed that high-glucose exposure elicited conspicuous ER damage—manifested as dilated ER cisternae, decreased ribosomal density, and disrupted ER membrane integrity—whereas MCU-i4 treatment substantially restored ER architecture.

Finally, the results of Western blotting (Figure 9g–j) indicated that MCU-i4 rescued high-glucose-impaired hippocampal synaptic plasticity, as evidenced by significant normalization of GAP43 and SYP levels (*p* < 0.05).

**Figure 10:**
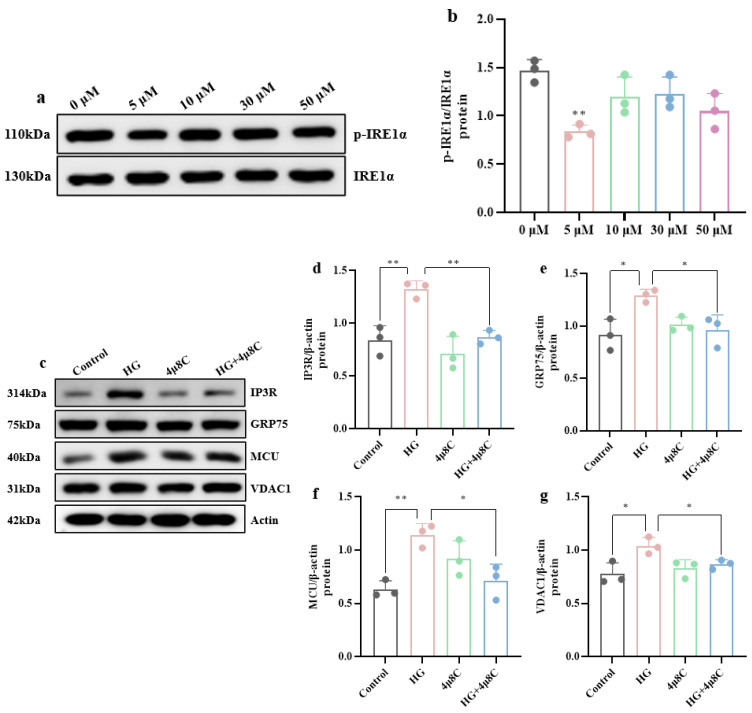

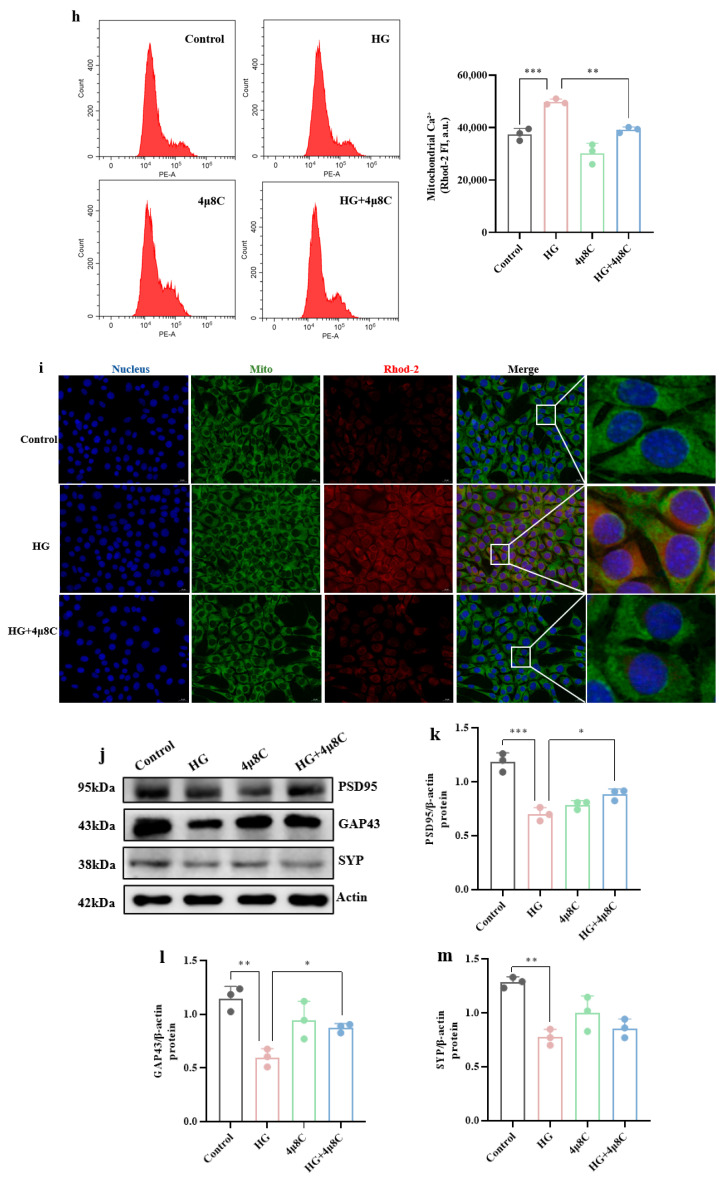
IRE1α pathway inhibition attenuates high-glucose-induced mitochondrial Ca^2+^ overload. (**a**) Western blot results of p-IRE1α and IRE1α protein expression. (**b**) Quantification of p-IRE1α/IRE1α protein expression. (**c**) Western blot results of Ca^2+^-transport protein expression. (**d**–**g**) Quantification of IP3R, GRP75, MCU, and VDAC1 protein expression. (**h**) Relative mitochondrial Ca^2+^ amounts determined by flow cytometry. (**i**) Fluorescence showing increased mitochondrial Ca^2+^ amounts in hippocampal neuronal cells exposed to high glucose and improvement after ERS inhibition. (Scale bar = 20 μm) (*n* = 3). (**j**) Expression levels of synaptic-related proteins. (**k**–**m**) Expression levels of PSD95, GAP43, and SYP protein in hippocampal neuronal cells. Data are presented as mean ± standard deviation. * *p* < 0.05, ** *p* < 0.01, *** *p* < 0.001.

We next applied 4μ8C to block IRE1α phosphorylation and suppress the ERS pathway. Western blotting (Figure 10a,b) showed that 5 μM 4μ8C significantly inhibited the IRE1α pathway (*p* < 0.01). When ERS was restrained, the high-glucose-induced overexpression of Ca^2+^-transport proteins in MAM was attenuated (Figure 10c–g): IP3R returned almost to control levels (*p* < 0.01), and GRP75, MCU, and VDAC1 were markedly reduced (*p* < 0.05). Flow cytometry (Figure 10h,i) revealed that the Rhod-2/AM fluorescence intensity in the HG+4μ8C group was markedly lower than that in the HG group (*p* < 0.01), indicating that inhibition of the IRE1α pathway effectively alleviated high-glucose-induced mitochondrial Ca^2+^ overload. Immunofluorescence (Figure 10j) further confirmed this observation: the number of colocalized puncta between Rhod-2/AM and the mitochondrial marker was substantially reduced after 4μ8C treatment.

Finally, Western blotting (Figure 10k–m) showed that 4μ8C-mediated inhibition of IRE1α phosphorylation restored synaptic protein levels, with PSD95 and GAP43 rising significantly (*p* < 0.05), indicating that ERS inhibition rescues high-glucose-induced hippocampal synaptic plasticity damage.

**Figure 11:**
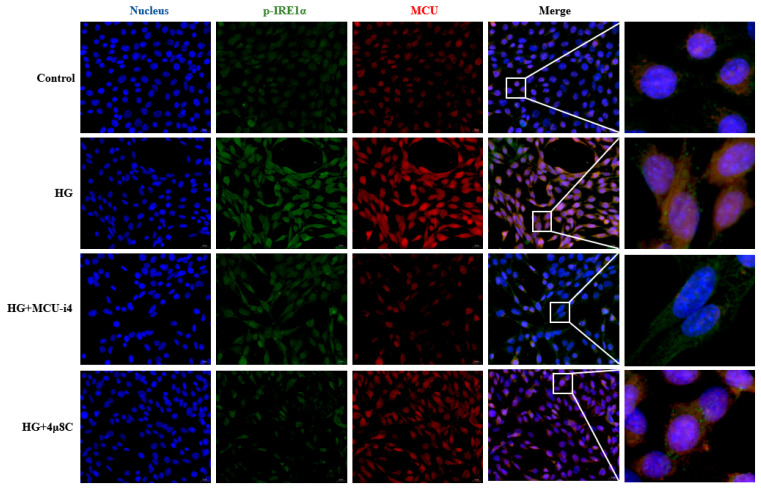
Immunofluorescence images of MCU and p-IRE1α proteins in hippocampal neurons (Scale bar = 20 μm) (*n* = 3).

Finally, to examine the interplay between MCU and p-IRE1α under high glucose, we performed immunofluorescence labeling. Figure 11 shows that exposure to 100 mmol/L glucose markedly increased MCU expression and intensified its fluorescent signal, while p-IRE1α levels and fluorescence were also elevated. Treatment with MCU-i4 to inhibit MCU expression attenuated the high-glucose-induced rise in p-IRE1α fluorescence, and application of 4μ8C to suppress IRE1α phosphorylation reduced the glucose-evoked increase in MCU fluorescence. These observations indicate a reciprocal regulatory relationship between MCU and p-IRE1α under high-glucose conditions.

## 4. Discussion

This is the first study to directly associate Mfn2-mediated MAM abnormalities with hippocampal synaptic plasticity injury in diabetic mice and to delineate the synergistic promotion of mitochondrial Ca^2+^ overload and ERS in this process, representing a novel target for diabetes-related cognitive impairment.

As is well known, diabetes is a chronic disease that severely affects human and animal health. With in-depth research, scientists have found that diabetes is closely related to neurodegenerative diseases such as Alzheimer’s disease and is one of the important causes of cognitive impairment [50]. Diabetes can induce neuroinflammation [51,52], cause neuronal apoptosis and necrosis [53,54], and activate autophagy [55,56,57], thereby causing neuronal damage and inducing cognitive impairment.

Our study found that diabetes-induced cognitive impairment in mice is accompanied by significant damage to synaptic structure and plasticity, which is consistent with previous studies. In the rat diabetes model established by Kang [58], the expression levels of synaptic-related proteins PSD95 and SYN1 were also significantly reduced. Gao [59] observed a significant decrease in the expression of synaptic-related proteins such as c-fos, Synapsin1, Synaptotagmin1, and BDNF in both mouse diabetes models and in vitro cultured hippocampal neuronal cell models, and melatonin can reverse the imbalance of these protein expressions. Qiu et al. [20] found that artemisinin may alleviate cognitive dysfunction in T2DM mice by activating the hippocampal PI3K/Akt pathway and enhancing synaptic plasticity. The aforementioned studies, along with the present research, consistently demonstrate that defects in hippocampal synaptic structure and function are common characteristics and significant pathological manifestations of cognitive dysfunction associated with diabetes.

MAM, the physical tethers between the endoplasmic reticulum and mitochondria, must maintain appropriate abundance and structural integrity to preserve cellular physiology [60]. Previous studies have found that dysregulation of MAM can trigger a series of pathological events, including perturbations in lipid metabolism [61,62], impairments in mitochondrial autophagy [63,64], and disruptions in calcium homeostasis [30,31], culminating in impairments of neuronal and synaptic functions. In our study, high-glucose increased MAM number, enlarged ER–mitochondria contact area, and elongated the functional interface in hippocampal neurons. Previous studies have demonstrated that Mfn2, a mitochondrial dynamin-related protein that bridges the endoplasmic reticulum and mitochondria, is enriched at the ER–mitochondria interface. Its loss disrupts ER morphology, loosens ER–mitochondria contacts, and reduces mitochondrial Ca^2+^ uptake efficiency [24]. Therefore, we knocked down Mfn2 to reverse these MAM aberrations and concurrently alleviated high-glucose-induced synaptic plasticity deficits, underlining the tight link between MAM dysfunction and synaptic impairment under high-glucose conditions. But how MAM regulates synaptic impairment under high-glucose conditions need further investigation.

Several investigators have identified Ca^2+^ dyshomeostasis—particularly mitochondrial Ca^2+^ overload—as the key event underlying glucose-induced neuronal dysfunction. Li Xu et al. [65] have found that under hypoxia, MAM Ca^2+^ homeostasis is disrupted, leading to mitochondrial Ca^2+^ overload and massive cytochrome C release that triggers the apoptotic program in photoreceptor cells. These findings are consistent with ours, as the application of the inhibitor MCU-i4 to block Ca^2+^ transport within MAM reduces high-glucose-induced mitochondrial Ca^2+^ overload in hippocampal nerve cells and improves high-glucose-induced synaptic plasticity impairment. In addition, Chang [32] found that in neuroblastoma, high-glucose-induced neuronal damage and mitochondrial Ca^2+^ overload can be alleviated by limiting the formation of abnormal MAM, thereby reducing neuroblastoma damage. Our results further indicate that Mfn2 knockdown alleviates high-glucose–evoked mitochondrial Ca^2+^ overload and consequently rescues synaptic plasticity damage. Thus, MAM modulates synaptic plasticity by regulating Ca^2+^ flux in hippocampal neurons.

ER stress likewise compromises neuronal integrity and synaptic plasticity. Gabriela Mercado et al. [66] suppressed the PERK pathway with the PERK inhibitor GSK2606414, to attenuate endoplasmic reticulum stress, restore dopamine levels and synaptic protein expression, and consequently alleviate neurodegeneration in Parkinson’s disease. Cao et al. [67] found that in podocytes of diabetic nephropathy mouse models, there is a decrease in Mfn2 expression, mitochondrial morphological abnormalities, and a reduction in the number of MAM, leading to ER stress and apoptosis, which in turn further affected the structure and function of synapses. Consistent with the above studies, we confirmed that high glucose activates ERS in hippocampal neurons, especially the IRE1α pathway, and that inhibiting its phosphorylation with the inhibitor 4μ8C alleviates high-glucose-induced synaptic plasticity injury. Interestingly, our data also reveal that suppressing ERS alleviates high-glucose-induced mitochondrial Ca^2+^ overload, whereas blocking Ca^2+^ flux at MAM attenuates high-glucose-induced ERS. Previous studies have already hinted at an intimate link between ERS and Ca^2+^ homeostasis. Bo Li et al. [68] reported that dibutyl phthalate activates ER stress to disrupt Ca^2+^ flux within MAM, thereby promoting NLRP3 inflammasome activation and pyroptosis that culminate in cardiac injury. Ji Yanwei et al. [69] further demonstrated that high glucose triggers ERS, provoking excessive Ca^2+^ transfer from the ER-to-mitochondria and inducing apoptosis in H9c2 cells, which they called the “ERS–MAM–mitochondrial Ca^2+^ overload” axis. Meanwhile, we observed a robust physical interaction between the ERS marker p-IRE1α and the MAM-located Ca^2+^ transporter MCU. Collectively, our findings reveal that the relationship is not a simple unidirectional axis but rather bidirectional: ERS perturbs Ca^2+^ homeostasis, while Ca^2+^ dyshomeostasis amplifies ERS. This mutual reinforcement underpins MAM-mediated synaptic plasticity impairment in high-glucose-exposed hippocampal neurons.

Nevertheless, several limitations remain to be addressed: The present findings are based on in vitro and ex vivo approaches. The absence of in vivo validation with Si-Mfn2, MCU-i4, and 4μ8C, together with the lack of direct electrophysiological recordings such as LTP/LTD, limits immediate translational interpretation. In follow-up projects, we will attempt to translate our current findings back into in vivo animal experiments for validation, aiming to confirm a novel target for diabetes-related cognitive impairment. In addition, our findings are derived from male mice only. Estrogen-related protection in females may alter the course of diabetes-associated cognitive decline. Future work should include both sexes to determine the generalizability of the observed neuroprotective mechanism.

## 5. Conclusions

Our study verified the cognitive dysfunction and synaptic plasticity damage in mice caused by diabetes, and that high glucose induces structural and numerical abnormalities of MAM and disturbs Ca^2+^ transport, leading to mitochondrial Ca^2+^ overload and ERS. These two processes then reciprocally reinforce one another to culminate in synaptic plasticity damage. These findings offer a previously unrecognized therapeutic avenue for preventing or reversing diabetic cognitive dysfunction.

## Figures and Tables

**Table 1:** Sequence of primers used in qRT-PCR.

Target Gene	Accession Number	Primer	Primer Sequence (5’-3’)
Mfn2	NM_001285920.1	Forward	GCTTGGACAGGTGGAGTCAA
		Reverse	GGACTCGAGGTCTCCTCTGT
PSD95	NM_001109752.1	Forward	AGCCCCAGGATATGTGAACG
		Reverse	ATGGAACCCGCCTCTTTGAG
SYP	NM_009305.2	Forward	GACGTTGGTAGTGCCTGTGA
		Reverse	GCACAGGAAAGTAGGGGGTC
GAP43	NM_008083.2	Forward	GATGGTGTCAAGCCGGAAGA
		Reverse	CCACGGAAGCTAGCCTGAAT
β-actin	NM_007393.5	Forward	TGTACCCAGGCATTGCTGAC
		Reverse	AACGCAGCTCAGTAACAGTCC

**Table 2:** RRIDs for antibodies.

Name of Antibody	RRID
Actin (ABclonal, Wuhan, China AC026)	AB_2768234
GAP43 (ABclonal, China A19055)	AB_2862548
Mfn2 (Proteintech, Wuhan, China 12186-1-AP)	AB_2266320
PSD95 (ABclonal, China A7889)	AB_2769180
SYP (ABclonal, China A19122)	AB_2862615
IP3R (ABclonal, China A4436)	AB_2314676
GRP75 (Wanleibio, China WL03209)	AB_1661203
MCU (ABclonal, China A22525)	AB_2902658
VDAC1 (ABclonal, China A19707)	AB_10564217
p-IRE1α (Nature Biosciences, Hangzhou, China A24402)	AB_3102214
IRE1α (Nature Biosciences, China A74484)	AB_2927490
XBP-1s (Nature Biosciences, China A58678)	AB_2737816

## Data Availability

The original contributions presented in this study are included in the article. Further inquiries can be directed to the corresponding author.

## Abbreviations

The following abbreviations are used in this manuscript:

MAM	Mitochondrial-associated endoplasmic reticulum membranes
T2DM	Type 2 diabetes mellitus
MWM	Morris water maze
TEM	Transmission electron microscopy
